# Mental health impacts of African swine fever outbreaks on veterinarians in the Philippines

**DOI:** 10.3389/fvets.2025.1519270

**Published:** 2025-03-11

**Authors:** Hannah J. Bakke, Alejandro D. Perez, Ruth Miclat-Sonaco, Andres M. Perez, Rachel A. Schambow

**Affiliations:** ^1^Center for Animal Health and Food Safety, College of Veterinary Medicine, University of Minnesota, Saint Paul, MN, United States; ^2^Department of Community Health, Dr. Armando Zamudio Hospital, Cmte. Luis Piedrabuena, Argentina; ^3^Department of Agriculture, National Livestock Program, Office of the Undersecretary for Livestock, Quezon City, Philippines

**Keywords:** African swine fever, mental health, public health, Philippines, questionnaire, One Health, pigs, social wellbeing

## Abstract

Emergence of African swine fever (ASF) in the Philippines in 2019 caused substantial impacts on animal health and its pig industry. To control ASF, strict policies were applied including zoning and depopulation of infected herds. While ASF’s severe impacts on pigs are well recognized, its potential impacts to public health are often overlooked. ASF is not a food safety concern and does not infect humans, but it has the potential to affect mental, emotional, and social well-being during emergency response. Veterinarians may be particularly at risk due to their role in depopulation of pigs and other distressing tasks. The objective here was to assess the effects of the ASF outbreaks on Filipino veterinarians’ mental and social well-being. A questionnaire was created and anonymously administered to 13 Filipino veterinarians attending a training workshop in December 2023. All participants had experience responding to the ASF outbreaks. Then, the summary responses were discussed with the entire group, allowing for clarification and verification. Two-by-two contingency tables and Fisher’s exact test were used to explore associations between responses. The top five negative signs reported by >50% participants were “reduced energy,” “reduced sleep,” “new feelings of hopelessness or sadness,” “new feelings of anger or frustration,” and “reduced enjoyment of life.” Some veterinarians also reported negative social interactions such as antagonism and blame toward public veterinarians. These results highlight the often-overlooked impacts of ASF on mental and social well-being and indicate the need for mental health support for veterinarians as part of comprehensive ASF mitigation efforts.

## 1 Introduction

African swine fever (ASF) is a complex, viral disease caused by the ASF virus (ASFV). ASFV only affects pigs and causes severe hemorrhagic symptoms, fever, and often high mortality nearing 100% of affected pigs (1–4). There is no treatment or vaccine for ASF, and the disease can only be prevented through strict biosecurity of pig farms. Because of its severity and significance to global pig health, ASF is reportable to the World Organisation for Animal Health (5). ASF was first described in Africa in 1921 and spread into Europe in 1957, resulting in the first widescale, global ASF epidemic (6). In 2007, a second wave of global ASF expansion began when the country of Georgia experienced an outbreak of the highly virulent genotype II ASFV strain. The ASFV was suspected of being spread from ASFV-contaminated pork in catering waste from ships that was fed to pigs (7). Since 2007, ASF has displayed transboundary and transcontinental spread across Europe and Asia, notably being detected in China in 2018 and the Dominican Republic and Haiti in 2021. This spread has made control more difficult and has given ASF global significance through its impacts to animal health and the global economy (3, 8–12).

ASF was detected in the Philippines in July 2019. These outbreaks caused significant disruption, including a 41.7% drop in pig production in 2021 and a 20.5% decrease in registered pigs in March 2023 compared to the same quarter in 2020 (13, 14). Approximately 5 million pigs in the Philippines have been culled due to ASF since 2019 which lead to significant economic impacts on farmers, consumers, and the economic stability of the country (14, 15). The ASF outbreaks continued to expand across the Philippines, to which the Filipino government responded by creating and implementing control programs, such as the National Zoning and Movement (NZM) plan for ASF in 2019 (16). This zoning is broken up into infected, buffer, surveillance, or protected zones. Culling of animals within 1 km from the infected zone and within 5 days or less was also implemented. Given the timing of the epidemic, corresponding with COVID-19 efforts and lack of resources and personnel, this program had varying degrees of success (14). There was pushback from pig stakeholders, however, given that pigs hold important economic value and personal and cultural significance in the Philippines (13). Unfortunately, and despite control efforts, ASF remains present in the Philippines, leading to continuing impacts on pig farmers and pigs, inflation of pork prices, and threatening the food security of vulnerable groups (17).

One Health has been defined as “an approach to address a health threat at the human-animal-environment interface based on collaboration, communication, and coordination across all relevant sectors and disciplines, with the ultimate goal of achieving optimal health outcomes for both people and animals” (18). Using the One Health lens in addressing ASF outbreaks allows for a broad view of the various factors affecting successful ASF control, and the recognition and evaluation of public health aspects of the ASF outbreaks such as food security, mental health, and social well-being. Social well-being refers to relationships and the way individuals interact with each other (19). Mental health refers to an individual’s emotional, psychological, and social well-being, and affects how they think, feel, act, relate to others, and handle stress (20). Stress is a physiological and psychological response of the body to situations perceived as demanding or threatening. Biologically, stress activates the sympathetic nervous system, releasing hormones such as cortisol and adrenaline, preparing us to face a challenge. It is a key adaptive mechanism for survival, but when persistent or excessive, it can have detrimental effects on physical and mental health. There are several subtypes of stress, classified according to their duration and the context in which they occur. Work stress is defined as the process by which workplace psychological experiences and demands (stressors) produce both short-term (strains) and long-term changes in mental and physical health and can be the result of an imbalance between job demands and available resources (21). Over time, chronic workplace stress can have negative impacts on an individual’s wellness and has been linked to burnout and depression (22).

Public and private veterinarians are key personnel in enacting ASF policies and regulations, such as diagnostic testing for regulatory purposes like movements and surveillance, and overseeing and conducting depopulation of ASF-positive herds. Their critical role may put them in a position to experience adverse mental and social effects from the ASF outbreaks. For example, culling pigs, particularly healthy animals to prevent further disease spread, can cause moral conflict for veterinarians whose role is typically to improve animal health. It could also create antagonism between them and private pig stakeholders. However, these and other potential effects of ASF response on veterinarians’ mental and social well-being have not yet been well evaluated or documented in previous works. The objective of this study was to assess the mental health and social well-being of veterinarians responding to the ASF epidemic in the Philippines. This work demonstrates the negative effects of ASF on veterinarians and provides important information for decision-makers that can be used to create support mechanisms for them during ASF response activities.

## 2 Materials and methods

This protocol was reviewed and approved by the University of Minnesota Institutional Review Board. To assess the effects of ASF on the mental health of Filipino veterinarians responding to ASF, a modified Delphi approach was used whereby individual responses were first anonymously collected, and then reviewed as a group to clarify and enhance interpretation of the findings (23). To gather individual responses, a questionnaire was written and administered in English, an official language of the Philippines, to collect information on mental and social wellbeing of veterinarians responding to ASF in the Philippines and compare between pre- and post-ASF outbreaks (Supplementary File 1). Questions were adapted from the World Health Organization Division of Mental Health Quality of Life assessment (24). A mixture of multiple choice and short answer questions were used. The questionnaire and methods were reviewed and approved by the University of Minnesota Institutional Review Board.

Questionnaire respondents were a convenience sample of veterinarians attending an in-person ASF training workshop at the Agricultural Training Institute-International Training Center for Pig Husbandry (ATI-ITCPH) in Batangas, Philippines, in December 2023. Briefly, these veterinarians were part of a cohort that was selected for a series of ASF training workshops by a joint committee of the Philippine College of Swine Practitioners, the Philippine Department of Agriculture Bureau of Animal Industry, and the ATI-ITCPH. The cohort participants were selected based on their years of experience (1–2 years minimum, 5–10 years preferred) and role in the pig industry, balancing the number of private and public veterinarians invited. This selection process took place in February and March 2023, independent of the study conducted here. The full training cohort (*n* = 25 veterinarians) had a median of 20 years of experience as veterinarians, and all had experience with ASF and had seen the disease in the field. The questionnaire was administered during one of the cohort’s training workshops in December 2023. Of the training cohort, 13 veterinarians attended the in-person workshop and therefore were able and eligible to participate in the questionnaire activity. All 13 trainees that were present completed the questionnaire.

The questionnaire was individually and anonymously completed by individuals at the in-person workshop under the facilitation of one of the co-authors (RAS). Prior to completing the questionnaire, the participants reviewed and signed a consent form, in conjunction with the requirements of the University of Minnesota Institutional Review Board. The questionnaire was administered anonymously and online using Qualtrics (25). After the participants completed the questionnaire, the preliminary aggregated responses were reviewed in an unstructured whole group discussion using the results feature of Qualtrics. Aggregated results were projected to the whole group, in a manner so that individual responses could not be identified as belonging to any particular person. Multiple choice questions were automatically displayed by Qualtrics as bar charts with counts and percentages, and open comment responses were displayed as text. For each question, the participants were invited to freely comment on the results with minimal prompting, including whether they had any confusion with the question, how they felt it did or did not generalize to other veterinarians and pig stakeholders in the Philippines, and anything else they felt was important to discuss. The co-author that was present moderated the discussion and captured notes electronically as the participants discussed each question.

The questionnaire data was analyzed using Statistical Analysis Software version 9.04 (SAS) (26). Frequency tables were produced that summarized the count and percentage for each categorical response. Two-by-two contingency tables were used to explore pairwise associations between the five most frequently mentioned signs. Significance was evaluated using Fisher’s exact test, and results were considered significant for *p*-values less than 0.05. Comments captured during the live whole group discussion were reviewed by the co-authors to further aid in interpretation of the findings.

## 3 Results

The five most commonly reported negative responses were “reduced energy,” “reduced sleep,” “new feelings of hopelessness or sadness,” “new feelings of anger or frustration,” and “reduced enjoyment of life” (Table 1; Supplementary File 2). Over half (*n* = 7) of respondents reported those five negative signs. Amongst the remaining respondents, two individuals reported four negative signs, one individual reported three negative signs, one individual reported two negative signs, one individual reported only one negative sign, and one individual reported no negative signs. Twelve participants (92%) reported that they felt positive about the future. None reported intrusive thoughts about death, dying, or that their family or community would be improved if they were gone, and none reported having started or increased visits to a mental health professional.

**Table 1:** Responses of yes/no mental and social-wellbeing questions from Filipino veterinarians that responded to African swine fever (ASF) outbreaks in the Philippines.

Question	Number of respondents with “Yes” response (%)
Do you feel positive about the future?	12 (92)
Have you experienced reduced energy since the ASF outbreak?	11 (84)
Have you experienced reduced enjoyment of life since the ASF outbreak?	10 (77)
Have you experienced new feelings of anger or frustration since the ASF outbreak?	10 (77)
Have you experienced reduced sleep since the ASF outbreak?	9 (69)
Have you experienced new feelings of hopelessness or sadness since the ASF outbreak?	9 (69)
Do you have trouble concentrating on tasks since the ASF outbreak?	6 (46)
Have you experienced poor memory since the ASF outbreak?	4 (31)
Have you experienced extreme changes in feelings of happiness and sadness since the ASF outbreak?	2 (15)
Do you have less self-worth or less confidence in yourself due to the ASF outbreak?	2 (15)
Have you had any intrusive thoughts about death or dying since the ASF outbreak?	0 (0)
Since the ASF outbreak, have you had any intrusive thoughts that your family or community would be improved if you were gone?	0 (0)
Have you started or increased your visits to a mental health professional since the outbreak?	0 (0)
Have you experienced any negative behaviors from your neighbors or social circle since the outbreak?	3 (23)
Do you still treat your neighbors the same pre-outbreak and post-outbreak?	11 (85)

**Figure 1:**
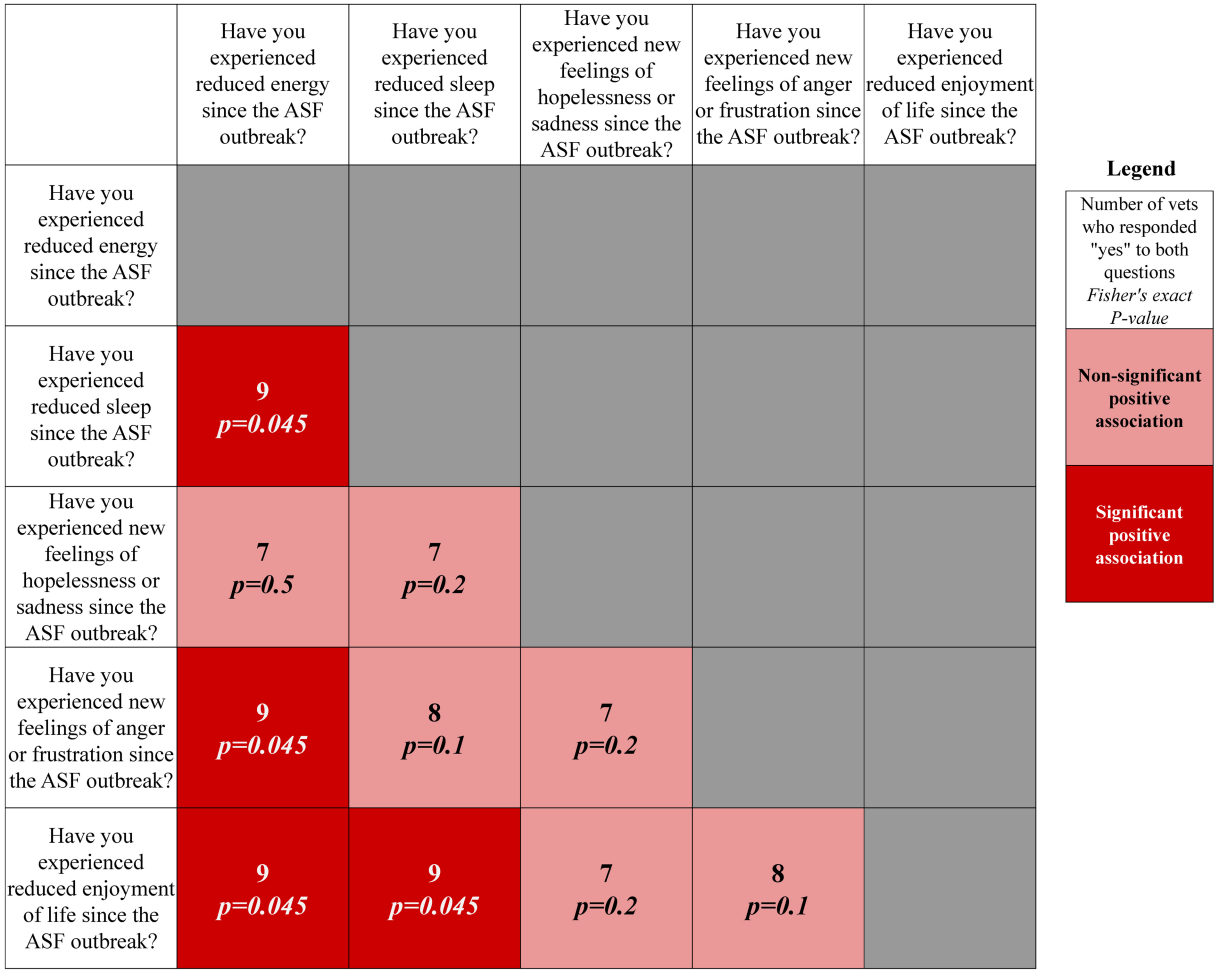
Correlation matrix of associations between responses to top reported mental health signs from Filipino veterinarians.

Four significant (*p* < 0.05), positive pairwise associations were found between the most commonly reported negative signs (Figure 1). Three of these related to “reduced energy,” including “reduced energy” and “reduced sleep”; “reduced energy” and “new feelings of frustration and anger”; and “reduced energy” and “reduced enjoyment of life.” Additionally, “reduced sleep” and “reduced enjoyment of life” were significantly and positively associated.

Considering aspects of their social-wellbeing and community involvement, the participants mainly reported increased involvement in the community (*n* = 6, 46%) or no change (*n* = 5, 38%). Only two reported less involvement (15%). The majority (*n* = 11, 85%) indicated that they still treated neighbors the same post-ASF outbreaks, and only three individuals reported that they experienced any negative behaviors from neighbors or social circles. In the comment field, these three reported negative social interactions such as avoiding interactions that they felt would lead to confrontations, and antagonism and blame toward public veterinarians because of farmers’ losses to ASF.

During the whole group discussion, the veterinarians described that activities such as depopulation of pig herds, surveillance and reporting cases in the midst of resistant farmers and producers, and imposing livestock movement restrictions and quarantine were stress-inducing events they had experienced. They reported that these results are likely generalizable to other public and private veterinarians involved in the ASF response, but that the mental health impact to pig farmers is likely worse because of the direct impacts of activities like herd depopulation to their livelihoods. They also discussed the limited availability and accessibility of mental health professionals in the Philippines.

## 4 Discussion

This study represents one of the first formal assessments of the negative impacts of ASF outbreaks on the mental health and social well-being of veterinarians. According to the veterinarians surveyed here, responding to the outbreaks caused new negative feelings and increased stress. Some participants also reported changes in their social interactions because of antagonism toward their roles as enacting government ASF policies. Generally, veterinarians responding to the outbreaks may experience guilt or fear of retribution. Guilt is closely related to ethics, morality, and personal responsibility and may arise when a person believes they have transgressed a moral principle or failed to fulfill an ethical duty (27, 28). Depopulation of whole herds of animals for disease control can conflict with a veterinarian’s desire to promote animal well-being, protect the food supply, and to support farmers, ultimately posing deep ethical and moral dilemmas for the individual (28).

These findings are consistent with reports of mental health impacts from other animal disease outbreaks, such as the foot-and-mouth disease (FMD) outbreaks in 2001 in the UK and in 2010 in Japan (29, 30). Previous studies have shown that, following disease outbreaks, post-traumatic stress disorder symptoms can appear as late as 1 year after the incident (31). Veterinarians in the FMD outbreaks had similar experiences to Filipino veterinary responders in the ASF outbreaks, including being exposed to repeated traumatic experiences such as culling herds (29, 30). Parallels between the FMD crisis and these current results of ASF response in the Philippines include loss of trust in authority, feelings of distress, and changes in social dynamics resulting in poor mental health, among others (29, 30). The experiences that Filipino veterinarians reported in responding to the ASF outbreaks are also consistent with those documented for human healthcare workers in responding to COVID-19. For example, behaviors associated with anxiety, depression, and sleep disturbances were similar between these groups (31). The changes in social behaviors reported by some of the respondents also parallel those shown by first responders in COVID-19 pandemic such as experiencing stigma, isolation, and social rejection (31, 32). Despite ASF not being a zoonotic epidemic, which would imply a greater fear of contagion for oneself or one’s loved ones, the consequences can be equally devastating for the health of veterinarians (33).

Significant associations between reduced energy, reduced sleep, new feelings of frustration and anger, and reduced enjoyment of life were also observed here. Sleep loss is closely related to emotional conditions such as anxiety and can disrupt emotional and cognitive functioning (34, 35). For veterinarians in emergency response, such as for ASF, increased time working and potential distress from tasks like animal depopulation may disrupt their regular sleeping patterns. Total or partial sleep deprivation has been significantly associated with increased anxiety levels (36, 37). Together, and if not managed, the negative interdependence of anxiety and lack of sleep can lead to significant emotional and cognitive consequences including increased vulnerability to depression and suicidality (35, 38, 39), emphasizing the need for effective management of both during emergency response.

The current study aimed to primarily document negative impacts, and therefore, potential positive impacts or experiences were not thoroughly captured or explored here. Despite reporting numerous negative feelings and increased stress, nearly all the participants responded that they still felt positive about the future. Arguably, it is possible that for some individuals, and under certain circumstances, there could be positive experiences associated with the ASF outbreaks. For example, in the 2001 FMD outbreaks in the UK, affected individuals reported that formal and informal support networks were a source of strength and helped to alleviate some suffering. In other disease and non-disease disasters and traumatic events, similar positive experiences have been reported, such as community closeness, increased resilience, and personal growth (40–42). However, these studies also highlight the complexity of individual responses to traumatic events. More work is needed to understand the variety and intensity of emotional and behavioral responses from exposure to animal health emergencies such as ASF.

This study had some important limitations and considerations. The surveyed population represented a convenience sample and the overall sample size was small, so these responses may not capture the wide range of experiences and viewpoints of Filipino veterinarians responding to ASF. Using a mainly closed-ended questionnaire provided a standardized way to collect individual responses within a limited timeframe (i.e., during a training workshop), but did not allow for participants to give open responses, which can limit the types of experiences captured. Interactive methods, such as interviews and focus groups, can be conducted in semi-structured ways with open-ended questions and with flexibility for follow-up questions between the researcher and participants (43). This can allow for a deeper exploration of participants’ expertise and experiences. Future research should utilize these techniques to capture a wide range of experiences during the ASF outbreaks, ultimately achieving greater qualitative data saturation and exploring the potential complexity that may be present (44). Additionally, the COVID-19 pandemic was occurring at the same time as the initial ASF outbreaks in the Philippines, and its effects may have exacerbated and/or masked the effects observed here from the ASF outbreaks. This study did not examine the effects on pig farmers, which require additional consideration and research. Pig farmers may face their own unique challenges to their mental and social well-being that would require additional support.

The ongoing ASF outbreaks in the Philippines have caused significant impacts on the country. Here, the negative impacts of ASF on veterinarians’ mental and social well-being were demonstrated, including reduced energy, reduced sleep, new feelings of anger and frustration, new feelings of hopelessness and sadness, and reduced enjoyment of life. These findings highlight the need to provide mental health training and preparedness for ASF responders to prevent and reduce negative mental and social impacts. Further work will help researchers and policymakers to understand the full vulnerabilities of at-risk communities such as veterinarians and allow for the creation of appropriate support mechanisms. Future studies should aim to further characterize ASF’s impacts on physical, mental, social, and economic wellbeing. Expanding this work to other ASF-affected countries will strengthen the ability to fully and accurately assess the public health impacts of an ASF outbreak and develop holistic mitigation strategies.

## Data Availability

The datasets presented in this article are not readily available because restrictions apply to the datasets: the datasets presented in this article are not readily available because of privacy and sensitivity concerns. Requests to access the datasets should be directed to Rachel Schambow, scham083@umn.edu.
