# Supplementary material for: Mental health impacts of African swine fever outbreaks on veterinarians in the Philippines
Source: Front Vet Sci. 2025 Mar 11;12:1519270. doi: 10.3389/fvets.2025.1519270 (PMC11933032; doi:10.3389/fvets.2025.1519270)
Supplement: Supplementary file 1 [file Table_1.docx]

Supplementary File 1. Questionnaire used to collect responses of mental and social wellbeing of veterinarians responding to ASF in the Philippines. Questionnaire was made and administered via Qualtrics.

Start of Block: Introduction

Welcome to the activities for today's workshop on African Swine Fever. Please begin by clicking the arrow in the bottom right of your screen.

In the following section, we aim to collect information about the public health impacts of ASF outbreaks on mental and social wellbeing.

**Mental & Behavioral Wellbeing:** How has mental and behavioral health been impacted since the ASF has occurred?

**Social Wellbeing:** How has society been impacted by the ASF outbreak?

Would you like to answer this section from your perspective as a veterinarian or farmer/producer? Please choose the most applicable role for you.

- Veterinarian
- Producer

End of Block: Public Health Section Intro

Start of Block: Public Health Mental and social wellbeing

Display This Question:

If Would you like to answer this section from your perspective as a veterinarian, farmer/producer, o... = Veterinarian

Have you attended field outbreaks of ASF?

- Yes
- No

Display This Question:

If Have you attended field outbreaks of ASF? = Yes

How many field outbreaks of ASF have you attended?

________________________________________________________________

**(Display this question if a producer/farmer)**

Did your farm experience an outbreak of ASF?

- Yes
- No

Is your physical health better or worse since the ASF outbreak?

- Better
- Worse
- No change

Have you experienced any changes in your physical exercise since the ASF outbreak?

- Exercise more
- Exercise less
- Exercise about the same

**Mental and Behavioral Wellbeing**
In the next section, we ask you to consider the impacts of ASF on mental, behavioral, and emotional wellbeing.

Have you experienced reduced energy since the ASF outbreak?

- Yes
- No

Have you experienced reduced sleep since the ASF outbreak?

- Yes
- No

Have you experienced reduced enjoyment of life since the ASF outbreak?

- Yes
- No

Do you feel positive about the future?

- Yes
- No

Have you experienced new feelings of hopelessness or sadness since the ASF outbreak?

- Yes
- No

Do you have trouble concentrating on tasks since the ASF outbreak?

- Yes
- No

Have you experienced poor memory since the ASF outbreak?

- Yes
- No

Have you experienced new feelings of anger or frustration since the ASF outbreak?

- Yes
- No

Have you experienced extreme changes in feelings of happiness and sadness since the ASF outbreak?

- Yes
- No

Do you have less self-worth or less confidence in yourself due to the ASF outbreak?

- Yes
- No

Have you had any intrusive thoughts about death or dying since the ASF outbreak?

- Yes
- No

Since the ASF outbreak, have you had any intrusive thoughts that your family or community would be improved if you were gone?

- Yes
- No

Has the ASF outbreak caused adverse physical health or emotional problems that have made it difficult for you to do social activities (such as visiting to friends or family)?

- Yes
- No
- No change

Have you started or increased your visits to a mental health professional since the outbreak?

- Yes
- No

| Page Break |  |
| --- | --- |

**Mental and Behavioral Wellbeing: Work and Employment**

Veterinarians only: How many farms did you visit per day pre-outbreak?

- Less than 1
- 1-3
- 4-5
- More than 5

Veterinarians only: How many farms do you visit per day post-outbreak?

- Less than 1
- 1-3
- 4-5
- More than 5

Have you lost work since the outbreak?

- Yes
- No

How has the time you spend working changed since the outbreak?

- Much less
- Somewhat less
- About the same
- Somewhat more
- Much more

How has your job satisfaction changed since the outbreak?

- Much worse
- Somewhat worse
- About the same
- Somewhat better
- Much better

| Page Break |  |
| --- | --- |

**Social Wellbeing**
In this final section, we ask you to consider topics related to your social wellbeing before and after an ASF outbreak.

Veterinarians only: Do you need to attend any Continuing Education courses?

- Yes
- No

Display This Question:

If Do you need to attend any Continuing Education (CE) courses? = Yes

Veterinarians only: Have you been able to continue those continuing education courses post-outbreak?

- Yes
- No

Do you have school-age children?

- Yes
- No

Display This Question:

If Do you have school-age children? = Yes

Have you had to remove your children from school due to bullying or ostracization due to an ASF outbreak?

- Yes
- No

Have you experienced any negative behaviors from your neighbors or social circle since the outbreak?

- Yes
- No

Display This Question:

If Have you experienced any negative behaviors from your neighbors or social circle since the outbreak? = Yes

What types of negative behaviors are your neighbors showing you and why do you think they are acting this way?

________________________________________________________________

How have your behaviors in society/your community changed since the outbreak(s)?

- More involved in community
- Less involved in community
- No change to community involvement
- Was never involved in community

Do you still treat your neighbors the same pre-outbreak and post-outbreak?

- Yes
- No

Display This Question:

If Do you still treat your neighbors the same pre-outbreak and post-outbreak? = No

What has changed, and why?

________________________________________________________________

Have you received any governmental involvement (positive or negative) due to the outbreak?

- Yes
- No

Display This Question:

If Have you received any governmental involvement (positive or negative) due to the outbreak? = Yes

What kind of involvement (financial/quarantine/fines/etc)? Check all that apply.

- Financial compensation
- Fines
- Quarantine/isolation
- Required by government to participate in depopulation on one of the farms I serve

Display This Question:

If Have you received any governmental involvement (positive or negative) due to the outbreak? = Yes

Was the involvement overall helpful or harmful to you/the community?

- Helpful
- Harmful

End of Block: Public Health Mental and social wellbeing

Start of Block: Thank you Conclusion

Thank you. You may use the back button to review your questions before submitting.

End of Block: Thank you Conclusion
