# Supplementary material for: Mental health impacts of African swine fever outbreaks on veterinarians in the Philippines
Source: Front Vet Sci. 2025 Mar 11;12:1519270. doi: 10.3389/fvets.2025.1519270 (PMC11933032; doi:10.3389/fvets.2025.1519270)
Supplement: Supplementary file 2 [file Table_2.docx]

Mental health impacts of African Swine Fever outbreaks on veterinarians in the Philippines

**Hannah J. Bakke^1^, Alejandro D. Perez^2^, Ruth Miclat-Sonaco^3^, Andres M. Perez^1,^ and Rachel A. Schambow^1*^**

Supplementary File 2. Responses to mental and social-wellbeing questionnaire from Filipino veterinarians that responded to African swine fever (ASF) outbreaks in the Philippines.

| **Question** | ***Questionnaire Response***  **Number (Percent of Respondents)** | | | | |
| --- | --- | --- | --- | --- | --- |
|  | ***Yes*** | | | | |
| Do you feel positive about the future? | 12 (92) | | | | |
| Have you experienced reduced energy since the ASF outbreak? | 11 (84) | | | | |
| Have you experienced reduced enjoyment of life since the ASF outbreak? | 10 (77) | | | | |
| Have you experienced new feelings of anger or frustration since the ASF outbreak? | 10 (77) | | | | |
| Have you experienced reduced sleep since the ASF outbreak? | 9 (69) | | | | |
| Have you experienced new feelings of hopelessness or sadness since the ASF outbreak? | 9 (69) | | | | |
| Do you have trouble concentrating on tasks since the ASF outbreak? | 6 (46) | | | | |
| Have you experienced poor memory since the ASF outbreak? | 4 (31) | | | | |
| Have you experienced extreme changes in feelings of happiness and sadness since the ASF outbreak? | 2 (15) | | | | |
| Do you have less self-worth or less confidence in yourself due to the ASF outbreak? | 2 (15) | | | | |
| Have you had any intrusive thoughts about death or dying since the ASF outbreak? | 0 (0) | | | | |
| Since the ASF outbreak, have you had any intrusive thoughts that your family or community would be improved if you were gone? | 0 (0) | | | | |
| Have you started or increased your visits to a mental health professional since the outbreak? | 0 (0) | | | | |
| Have you experienced any negative behaviors from your neighbors or social circle since the outbreak? | 3 (23) | | | | |
| Do you still treat your neighbors the same pre-outbreak and post-outbreak? | 11 (85) | | | | |
| Have you lost work since the outbreak? | 2 (15) | | | | |
| Do you need to attend any continuing education courses? | 11 (79) | | | | |
| Have you been able to continue your continuing education courses post-outbreak? | 8 (73% of those who answered "Yes" to needing to attend continuing education courses) | | | | |
| Do you have school-age children? | 5 (42) | | | | |
| Have you had to remove your children from school due to bullying or ostracization due to an ASF outbreak? | 0 (100% of those with school-age children) | | | | |
| Have you received any governmental involvement (positive or negative) due to the outbreak? | 10 (76) | | | | |
| How have your behaviors in society/your community changed since the outbreak(s)? | ***Less involved*** | ***No change*** | | ***More involved*** | |
|  | 2 (15) | 5 (38) | | 6 (46) | |
| Have you experienced any changes in your physical exercise since the ASF outbreak? | ***Less*** | ***About the same*** | | ***More*** | |
|  | 4 (31) | 7 (54) | | 2 (15) | |
| Is your physical health better or worse since the ASF outbreak? | ***Worse*** | ***No Change*** | | ***Better*** | |
|  | 4 | 8 | | 0 | |
| Has the ASF outbreak caused adverse physical health or emotional problems that have made it difficult for you to do social activities? | ***No*** | ***No Change*** | | ***Yes*** | |
|  | 4 (31) | 6 (46) | | 3 (23) | |
| How many farms did you visit per day pre-outbreak? | ***Less than 1*** | ***1 to 3*** | ***4 to 5*** | ***More than 5*** | |
|  | 6 (46) | 6 (46) | 0 (0) | 1 (8) | |
| How many farms do you visit per day post-outbreak? | 7 (54) | 3 (23) | 0 (0) | 3 (23) | |
| How has the time you spend working changed since the outbreak? | ***Much less*** | ***Somewhat less*** | ***About the same*** | ***Somewhat more*** | ***Much more*** |
|  | 0 | 2 | 2 | 3 | 6 |
| How has your job satisfaction changed since the outbreak? | ***Much worse*** | ***Somewhat worse*** | ***About the same*** | ***Somewhat better*** | ***Much better*** |
|  | 1 (8) | 3 (23) | 7 (54) | 2 (15) | 0 (0) |
| What kind of involvement (financial/quarantine/fines/etc? Check all that apply. | ***Fines*** | ***Financial Compensation*** | ***Quarantine and/or isolation*** | ***Required by government to participate in depopulation on one of the farms I serve*** | |
|  | 2 (20)* | 0 (0)* | 6 (60)* | 6 (60)* | |
| Was the involvement overall helpful or harmful to you/the community? | ***Harmful*** | | | ***Helpful*** | |
|  | 4 (40)* | | | 6 (60)* | |
